# 2313. Population-based Incidence of SARS-CoV-2-associated Hospitalizations across the Age Spectrum in New York City, 2020-2021

**DOI:** 10.1093/ofid/ofad500.1935

**Published:** 2023-11-27

**Authors:** Lisa Saiman, Edward E Walsh, Angela R Branche, Angela Barrett, Luis R Alba, Sonia Gollerkeri, Julia A Schillinger, M A T T H E W R PHILLIPS, Lynn Finelli

**Affiliations:** Columbia University Irving Medical Center, New York, NY; University of Rochester, Rochester, NY; University of Rochester, Rochester, NY; Columbia University Irving Medical Center, New York, NY; Columbia University Medical Center, New York, New York; Columbia Irving Medical Center, Dept. Pediatrics, New York, New York; Merck & Co., Inc., Rahway, New Jersey; Merck & Co., Inc., Rahway, NJ, USA, PHILADELPHIA, Pennsylvania; Merck&Co., Rose Valley, Pennsylvania

## Abstract

**Background:**

Population-based incidence rates for hospitalizations due to SARS-CoV-2 can provide an increased understanding of the impact of transmission and mitigation strategies over time, yet there are relatively few estimates of such rates. Thus, we conducted a population-based, surveillance study to estimate incidence rates of SARS-CoV-2 hospitalizations among patients across the age spectrum.

**Figure:**
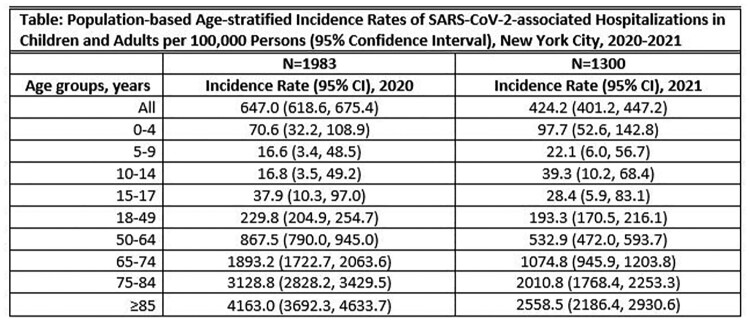

**Methods:**

From March 2020 to December 2021, we retrospectively reviewed the electronic medical records of patients admitted to an academic, multi-hospital system in New York City (NYC) to determine population-based incidence rates. Eligible patients resided in the pre-defined surveillance area, had ≥2 acute symptoms, and had laboratory-confirmed SARS-CoV-2 infection detected within 14 calendar-days prior to admission to 2 calendar-days after admission. Overall and age group incidence of SARS-CoV-2 hospitalization per 100,000 persons living in the surveillance area were calculated using 2020 census data, adjusted by hospital market share.

**Results:**

From 2020-2021, 3,283 eligible patients were hospitalized with SARS-CoV-2 at the study hospitals. Patients’ median age in 2020 was 69 years (IQR 56, 80) and in 2021 was 67 years (IQR 51, 79) and 2.5% were 0-17 years old. Overall, 52% were male and 11%, 10%, and 61% were Black, White, and Hispanic, respectively. An estimated 1-4% of adults in age groups >65 years living in the surveillance area were hospitalized (Table). From 2020 to 2021, the overall population-based annual incidence rate and annual incidence rates in age groups 50-64, 65-74, 75-84, and ≥85 years decreased. While not significant, the incidence rate increased in patients 0-14 years.

**Conclusion:**

These population-based incidence rates provide unique insights into the SARS-CoV-2 burden in a predefined surveillance area in NYC which served as the epicenter of the first U.S. wave of the pandemic. While incidence rates decreased in those >50 years old, rates were largely unchanged in those < 50 years old suggesting that younger adults and children had continued and potentially increased exposures to SARS-CoV-2 and/or lower vaccination rates than older adults during the second full year of the pandemic. Such incidence data can measure the impact of mitigation strategies.

**Disclosures:**

**Lisa Saiman, MD MPH**, Merck & Co., Inc,: Grant/Research Support|Merck & Co., Inc,: Member, DSMB|Pfizer, Inc: Member, DSMB **Edward E. Walsh, MD**, Icosavax: Advisor/Consultant|Merck: Advisor/Consultant|Merck: Grant/Research Support|Merck: Honoraria|Moderna: Advisor/Consultant|Pfizer: Grant/Research Support **Angela R. Branche, MD**, Cyanvac: Grant/Research Support|GSK: Advisor/Consultant|Janssen: Advisor/Consultant|Merck: Grant/Research Support|Pfizer: Grant/Research Support **Julia A. Schillinger, MD, MSc**, Merck & Co.: Employee, Stocks and Bonds **MATTHEW R. PHILLIPS, MPH**, Merck & Co., Inc., Rahway, NJ, USA: Employee|Merck & Co., Inc., Rahway, NJ, USA: Stocks/Bonds **Lynn Finelli, DrPH, MS**, Merck&Co: Stocks/Bonds

